# Predicting postoperative surgical site infection with administrative data: a random forests algorithm

**DOI:** 10.1186/s12874-021-01369-9

**Published:** 2021-08-28

**Authors:** Yelena Petrosyan, Kednapa Thavorn, Glenys Smith, Malcolm Maclure, Roanne Preston, Carl van Walravan, Alan J. Forster

**Affiliations:** 1 Clinical Epidemiology, Ottawa Hospital Research Institute, 1053 Carling Ave, Ottawa, Ontario K1Y 4E9 Canada; 2 School of Epidemiology and Public Health, University of Ottawa, 75 Laurier Ave E, Ottawa, Ontario K1N 6N5 Canada; 3 Institute for Clinical and Evaluative Sciences, 1053 Carling Ave, Ottawa, Ontario K1Y 4E9 Canada; 4 The Ottawa Hospital - General Campus, 501 Smyth Road, PO Box 201B, Ottawa, ON K1H 8L6 Canada; 5 Department of Anesthesiology, Pharmacology and Therapeutics, University of British Columbia, Vancouver, British Columbia V6T 1Z4 Canada; 6 Department of Medicine, University of Ottawa, 75 Laurier Ave E, Ottawa, Ontario K1N 6N5 Canada

**Keywords:** Surgical site infection, Administrative data, Machine learning, Random forests, Data mining, Predictive modeling

## Abstract

**Background:**

Since primary data collection can be time-consuming and expensive, surgical site infections (SSIs) could ideally be monitored using routinely collected administrative data. We derived and internally validated efficient algorithms to identify SSIs within 30 days after surgery with health administrative data, using Machine Learning algorithms.

**Methods:**

All patients enrolled in the National Surgical Quality Improvement Program from the Ottawa Hospital were linked to administrative datasets in Ontario, Canada. Machine Learning approaches, including a Random Forests algorithm and the high-performance logistic regression, were used to derive parsimonious models to predict SSI status. Finally, a risk score methodology was used to transform the final models into the risk score system. The SSI risk models were validated in the validation datasets.

**Results:**

Of 14,351 patients, 795 (5.5%) had an SSI. First, separate predictive models were built for three distinct administrative datasets. The final model, including hospitalization diagnostic, physician diagnostic and procedure codes, demonstrated excellent discrimination (C statistics, 0.91, 95% CI, 0.90–0.92) and calibration (Hosmer-Lemeshow χ^2^ statistics, 4.531, *p* = 0.402).

**Conclusion:**

We demonstrated that health administrative data can be effectively used to identify SSIs. Machine learning algorithms have shown a high degree of accuracy in predicting postoperative SSIs and can integrate and utilize a large amount of administrative data. External validation of this model is required before it can be routinely used to identify SSIs.

**Supplementary Information:**

The online version contains supplementary material available at 10.1186/s12874-021-01369-9.

## Background

Surgical site infection (SSI) is common and considered one of the most common types of postoperative complications [1]. SSIs are associated with substantial morbidity and mortality, prolonged hospital duration of stay, increased hospital readmission rate, and financial burden to health care systems [1–5]. Previous research has shown the importance of effective prevention strategies targeting both short- and long-term consequences of SSI, which requires an ability to track SSIs [2]. Since the primary data collection can be time-consuming and expensive, routinely collected health administrative data offer ample opportunities to identify and monitor SSIs, and assess the impact of prevention strategies, given a wide population coverage and minimal costs and efforts. Several studies have developed some accurate administrative algorithms to identify SSIs [6–10], while other studies have found that SSI identification using administrative data is imprecise [11]. However, previous studies were often based on small sample sizes and/or a limited set of pre-selected variables to predict SSIs.

Machine learning approaches have been successfully applied to create predictive models in several fields of study, including automatic medical diagnostics [12, 13]. With interpretability of model parameters and ease of use, logistic regression can generate excellent models and serve as a commonly accepted statistical tool. Random Forests approach is used in situations where regression assumptions may be violated by situations in which many predictors are associated with a small number of outcomes [14]. It can cope with inter-correlation between multiple explanatory variables, since each predictor is selected randomly for each stage of the learning process [15], unlike standard regression approaches. Previous studies have indicated that the Random Forests approach may have better prediction accuracy than other machine learning methods [16, 17]. We hypothesized that the use of machine learning approaches and a large data set with many features will improve the accuracy of SSI prediction. This study aimed to develop efficient algorithms to identify SSIs within 30 days after surgery using health administrative data.

### Material and methods

This study was divided into three stages. In the first stage, a Random Forests algorithm was used to perform a preliminary screening of variables and to rank the importance of candidate variables. In the second stage, the 30 most important variables from the first stage were input into the high-performance logistic regression to build interpretable and parsimonious models for all three administrative datasets used in this study. Finally, we used risk score modeling methodology to transform the final logistic models form the second stage into the risk score system.

### Selection and description of participants

This study was performed at The Ottawa hospital (TOH), Canada, a 1200-bed academic health sciences center providing approximately 90% of the major surgical operations in a catchment area of 1.2 million people. We identified all patients at TOH aged 18 years and older who underwent surgery and were included in the American College of Surgeons National Surgical Quality Improvement Program (NSQIP) data collection, between April 1, 2010, and March 31, 2015. The NSQIP uses trained Surgical Clinical Reviewers to collect data using a combination of chart review and follow up from the preoperative period through 30 days postoperatively. Patients were excluded if: 1) they were not eligible for the Ontario Health Insurance Program (OHIP) or had an invalid OHIP number, because this was required for linkage to health administrative datasets; or 2) they had missing admission, discharge, or surgery dates.

### Population-based health administrative datasets

We linked the NSQIP dataset to three distinct population-based, health administrative datasets housed at the Institute for Clinical and Evaluative Sciences (ICES). ICES is an independent, non-profit research institute whose legal status under Ontario’s health information privacy law allows it to collect and analyze health care and demographic data, without informed consent, for health system evaluation and improvement. The use of data in this project was authorized under section 45 of Ontario’s Personal Health Information Protection Act, which does not require review by a Research Ethics Board. The datasets included: 1) the Discharged Abstract Database and Same Day Surgery Database to identify the records of the hospitalization (ICD-10 code), including admission and discharge dates, diagnoses, 2) the Physician Services Database to retrieve all claims for services provided by all eligible health care providers, and 3) the Ontario Health Insurance Plan (OHIP) database that contains physician diagnostic codes (ICD-9 codes) and diagnosis descriptions. All patients were followed for 30 days from the time of their surgery. All databases were linked using anonymized unique identifiers and analyzed at the ICES at the University of Ottawa, Ontario. This study was approved by the Ottawa Health Science Network Research Ethics Board.

### Study outcome

All individuals who had any type of SSIs (i.e. superficial, deep, or organ space) (Additional file 1) within 30 days after surgery, according to the definition of the NSQIP protocol, were defined as having experienced an SSI.

### Statistical analysis

This study utilized a 3-stage predictive modeling based on the hybrid modeling approaches developed in previous studies [14, 15, 18]. All stages described below were applied to each administrative dataset used in this study to generate three sub-models that contributed to the omnibus SSI model.

### Stage 1 – model development using random forests algorithm

Details of Random Forests method have been described elsewhere [19–21]. In short, each of the classification trees is built using a bootstrap sample of the data, and a random subset of variables was selected at each split, thereby constructing a large collection of decision trees with controlled variation [22, 23] (Additional file 2). The Random Forests trees are not pruned, so as to obtain low-bias trees. Every tree in the forest casts a “vote” for the best classification for a given observation, and the class receiving most votes results in the prediction for that observation. The study cohort was first divided randomly into derivation (70%) and validation (30%) samples (Additional file 3). Then, the derivation data was sampled to create an in-bag partition – (2/3) to construct the decision tree, and a smaller out-of-bag partition (1/3) to test the constructed tree to evaluate its performance by computing: 1) misclassification error, 2) C-statistics, and 3) model performance (sensitivity, specificity, etc.). The optimal number of trees and a subset of variables at each node were selected using the “tuneRF” function in R to minimize the misclassification error. Random Forests calculates estimates of variable importance for classification using permutation variable importance measure (VIM) [19], which is based on the decrease of a classification accuracy when values of a variable in a node of a tree are permuted randomly. Finally, K-fold cross validation was used to evaluate the Random Forests model with 10 folds. We identified subsets of top 30 important diagnostic or procedure codes to predict SSIs, using a mean decrease accuracy value of 0.02 as a cut-off point. The Random Forests analyses were performed in R statistical software (3.3.2.) using “randomForest” package [21].

### Stage 2 – stepwise model selection using high-performance logistic regression approach

Random forests algorithm was used to perform a preliminary screening of variables and to gain importance ranks. Then, the selected top-30 important predictors were input into the high-performance logistic model with stepwise variable selection to find the best parsimonious model to predict SSIs [14, 24, 25]. High-performance logistic regression (proc hplogisitc) belongs to the high-performance analytics procedures that can be used to reduce the dimension or identify important variables to obtain parsimonious predictive models [26]. It permits several link functions and can handle ordinal and nominal data with more than two response categories [26]. The Schwarz Bayesian Criterion (SBC) was used as a penalized measure of fit for logistic regression model to help avoid the model over-fitting.

### Stage 3 – point system or risk scores

We used the methods suggested by Sullivan et al. [27] to summarize each logistic model from stage 2 as a point system. The point system or risk scores provide statistical information in a more clinically useful form than logistic regression models, as generalizability of the models developed from data from a single or a small group of hospitals to other patient populations is questionable [28, 29]. Clinical prediction models and associated risk-scoring systems are popular statistical methods as they permit a rapid assessment of patient risk without the use of computers or other electronic devices [30]. The use of such points-based systems facilitates evidence-based clinical decision making [30]. The point system developed in this study was designed to predict the risk of postoperative SSIs, based on a patient’s pre-procedural risk factors or predictors. The point score assigned to each predictor was derived from a well-fit logistic regression model.

The point scores were developed for hospitalization (ICD-10) and physician (ICD-9) diagnostic codes, and physician procedure claims. All variables in the models were categorical, and the distance between a variable and its base category in regression coefficient units was equal to the size of the coefficient. For each variable, its distance from the base category in regression coefficient units was divided by this constant and rounded to the nearest integer to get its point value.

Then, the obtained point scores were input into logistic regression model and adjusted for other potential confounding factors suggested by the existing literature, including age, sex, surgical procedure, emergency case, concurrent surgical procedures, patient’s physical status (ASA-5), and duration of surgery. The full model discrimination (C statistics or AUC) and calibration (Hosmer-Lemeshow (H-L) statistics) were assessed in the validation dataset. All methods were performed in accordance with the guidelines for developing and reporting Machine Learning predictive models in biomedical research [31]. The high-performance regression and point score assignment were performed in SAS 9.4 statistical software.

## Results

We identified 14,351 patients who underwent surgery from April 1, 2010 to March 31, 2015 and were enrolled into NSQIP at our hospital. An SSI was identified in 795 (5.5%) of these patients. Of these, 540 (68%) had superficial SSIs and 255 (32%) had deep or organ space SSIs. Descriptive statistics for patients in the study sample are reported in Additional file 4. The derivation and validation datasets were similar in terms of baseline covariates (Additional file 5).

### Predictive modeling for hospitalization diagnostic codes (ICD-10)

We identified 3085 hospitalization diagnostic (ICD-10) codes recorded within 30 days following the surgery date. These codes then were clustered into 994 three-digit hospitalization diagnostic codes that were used for the further analyses.

**Fig. 1:**
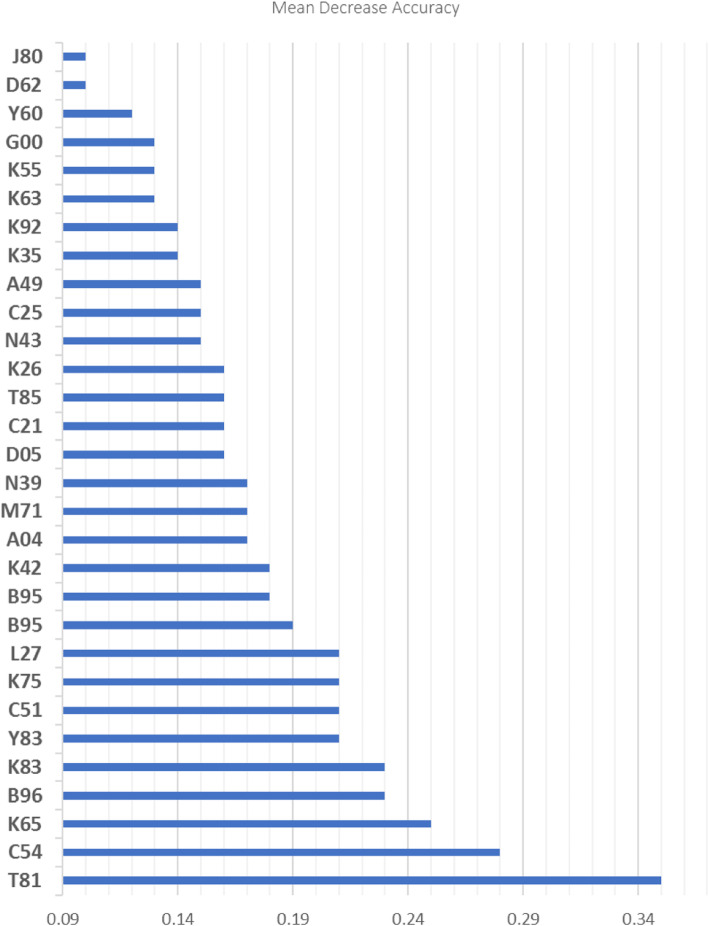
Description of top 30 hospitalization diagnostic (ICD-10) codes to identify SSIs. T81 – Operative complication (infection, hemorrhage, etc.); C54 – Malignant neoplasm of specified part of uterus; K65 – Peritonitis; B96 - Other bacterial agents as the cause of diseases classified elsewhere; K83 – Biliary duct infection, obstruction, perforation, or fistulation; Y83 - Surgical operation/procedures as the cause of abnormal reaction of the patient/or later complication; C51 - Malignant neoplasms of female genital organs; Y83 - Surgical operation/procedures as the cause of abnormal reaction of the patient/complication; C51 - Malignant neoplasms of female genital organs; K75 - Abscess of liver; L27 - Dermatitis and eczema; B95 - Streptococcus and staphylococcus as the cause of diseases; K42 - Umbilical hernia; A04 - Other bacterial intestinal infections; M71 – Bursal abscess, cyst, infection; N39 - Other disorders of urinary system; D05 - Carcinoma in situ of breast; C21 - Malignant neoplasm of anus and anal canal; T85 - Complications of internal prosthetic devices, implants and grafts; K26 - Duodenal ulcer; N43 - Other disorders of prostate; C25 - Malignant neoplasm of pancreas; A49 - Bacterial infection of unspecified site; K35 - Acute appendicitis; K92 - Other diseases of digestive system; K63 – Other diseases of intestine; K55 - Vascular disorders of intestine; G00 - Bacterial meningitis, unspecified; Y60 - Unintentional cut, puncture, perforation or haemorrhage during surgical and medical care; D62 - Acute posthaemorrhagic anemia; J80 - Acute respiratory distress syndrome

Stage 1: Given a large number of diagnostic codes (possible predictors), the Random Forests approach was used to identify a subset of top important 30 hospitalization diagnostic codes that best predicts classification. We used 800 classification trees and 46 variables available for splitting at each tree node. The accuracy of the Random Forests model was 95.3%. The resulting SSI prediction model demonstrated positive predictive value (PPV) of 98%, negative predictive value (NPV) of 97%, and AUC (area under the receiver operating characteristic curve) of 0.78 (95% CI 0.77–0.79). The accuracy of the Random Forests model after a 10-fold cross-validation was 94.3%. Figure 1 presents the top 30 hospitalization diagnostic (ICD-10) codes for classification of SSIs that have been identified using the permutation VIM.

Stage 2: The identified top 30 hospitalization diagnostic codes (ICD-10) codes were input into the high-performance logistic regression with a stepwise selection to identify the best parsimonious model to predict SSIs. Table 1, model 1 presents the final model of six hospitalization diagnostic codes to identify SSIs (AUC 0.87, 95% CI 0.86–0.89).

**Table 1:** The best parsimonious models for prediction of SSIs

**Model 1. The best parsimonious model of hospitalization diagnostic (ICD 10) codes**		
Effect	^*^AOR, 95% CI	Risk point
**T81-** Operative complication (infection, hemorrhage, etc.)	6.40 (5.08–8.01)	2
**K65 -** Peritonitis	5.87 (3.88–7.88)	1
**B96 -** Other bacterial agents causing infections	2.56 (1.84–3.47)	1
**K83 -** Biliary duct infection, obstruction, perforation	6.32 (4.42–8.01)	3
**Y83** - Surgical operation/procedures as the cause of abnormal reaction of the patient/ or later complication	2.46 (1.97–3.07)	1
**B95 –** Streptococcus/ staphylococcus as the cause of diseases	3.25 (2.17–4.87)	1
**Model 2. The best parsimonious model of physician diagnostic (ICD 9) codes**		
Effect	AOR, 95% CI	Risk point
**686** - Pyoderma, pyogenic granuloma, other local infections	8.13 (6.50–9.20)	3
**682-** Cellulitis, abscess	4.70 (3.57–6.10)	2
**998 -** Other complications of procedures	5.68 (4.77–6.78)	2
**556 -** Ulcerative colitis	8.60 (6.31–9.18)	3
**685 -** Pilonidal cyst with fistula, abscess	2.69 (1.52–3.76)	2
**560 -** Intestinal obstruction without mention of hernia	2.97 (2.19–4.01)	2
**154 -** Malignant neoplasm of rectum, rectosigmoid junction	4.37 (3.29–5.17)	2
**599 -** Other disorders of urethra and urinary tract	2.04 (1.55–2.62)	1
**153 -** Malignant neoplasm of colon	2.71 (2.02–3.22)	1
**Model 3. The best parsimonious model of physician procedure claims**		
Effect	AOR, 95% CI	Risk point
**Z59 -** Digestive system surgical procedure: colon/biliary tract	7.38 (6.08–9.09)	4
**C46 -** Infectious disease: hospital consult/assessment	5.77 (4.66–7.43)	3
**Z10 -** Skin/subcutaneous tissue: incision of abscess or hematoma	7.88 (6.04–8.67)	3
**C03** - General surgery: hospital consult/assessment	3.45 (2.86–4.19)	2
**H15 -** Family practice: assessment on weekend	2.33 (1.80–3.01)	2
**S16 -** Digestive system surgical procedures: intestine	1.98 (1.48–2.52)	1
**C20 -** Obstetrics and gynecology assessment/consult	2.25 (1.66–3.05)	2
**Z08 -** Debridement of wound(s) and/or ulcer(s)	4.01 (2.83–5.56)	3
**S21 -** Digestive system surgical procedures: colon/rectum	2.65 (1.91–3.62)	2
**R06 -** Skin/subcutaneous tissue: free island flaps	4.64 (2.58–6.36)	3
**C13 -** Internal medicine: hospital assessment/consult	1.96 (1.52–2.36)	1
**H13 -** Family practice: assessment/consult on weekdays	2.85 (2.18–3.52)	2
**C21 –** Pain management: limited consultations	1.84 (1.55–2.10)	1
**R11-** Operations of the breast: incision, excision, repair	2.81 (1.02–3.41)	3

^*^AOR, 95% CI = Adjusted Odds Ratio, 95% Confidence Interval

Stage 3: Risk scores for the final model of hospitalization diagnostic (ICD-10) codes are presented in Table 1, Model 1 [27]. Among the entire cohort, 80.3% of patients had a score of 0, 11.8% had a score of 1, and 7.9% had a score equal or greater than 2.

*Predictive modeling for physician diagnostic (ICD-9) codes.*

We identified 442 physician diagnostic 3-digit codes (using ICD-9-CA) recorded within 30 days following the surgery date.

**Fig. 2:**
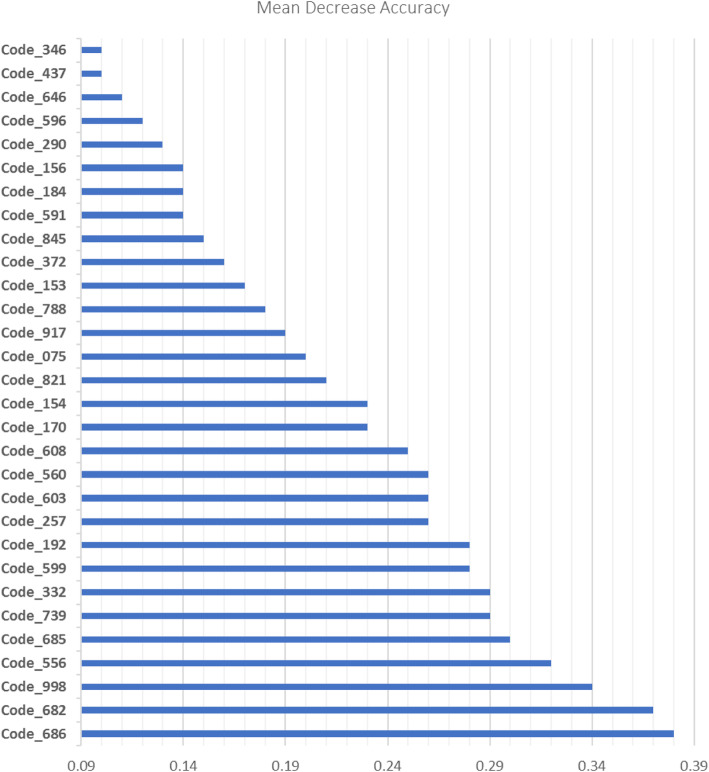
Description of the top 30 physician diagnostic (ICD-9) codes to identify SSIs. 686 - Pyoderma, pyogenic granuloma, other local skin infections; 682 - Cellulitis, abscess; 998 - Other complications of procedures, not elsewhere classified; 556 - Ulcerative colitis; 685 - Pilonidal cyst or abscess; 739 - Nonallopathic lesions, not elsewhere classified; 332 - Parkinson’s disease; 599 - Other disorders of urethra and urinary tract; 192 - Malignant neoplasm of other and unspecified parts of nervous system; 257 - Testicular dysfunction; 603 – Hydrocele; 560 - Intestinal obstruction without mention of hernia; 608 - Other disorders of male genital organs; 170 - Malignant neoplasm of bone and articular cartilage; 154 - Malignant neoplasm of rectum, rectosigmoid junction and anus; 821 - Fracture of femur; 075- Infectious mononucleosis, glandular fever; 917- Superficial injury of foot and toe(s); 788 - Symptoms involving urinary system; 153 – Malignant neoplasm of large intestine - excluding rectum; 372 - Conjunctiva disorders (e.g., conjunctivitis, pterygium); 845 – Sprains and strains of ankle and foot; 591 – Hydronephrosis; 184 - Malignant neoplasm of vagina, vulva, other female genital organs; 156 - Malignant neoplasm of gallbladder and extra hepatic bile ducts; 290 - Senile dementia, presenile dementia; 569- Other disorders of intestine; 646 - Other complications of pregnancy (e.g., vulvitis, vaginitis, cervicitis, pyelitis, cystitis); 437- Other and ill-defined cerebrovascular disease; 346 - Other diseases of central nervous system (e.g., brain abscess, narcolepsy, motor neuron disease, syringomyelia)

Stage 1: Given a large number of diagnostic codes (possible predictors), the Random Forests approach was used to identify a subset of 30 physician diagnostic codes that best predicts SSIs. The best misclassification rate was achieved by using 800 classification trees and 31 variables available for splitting at each tree node. The accuracy of the Random Forests model was 94.7%. The resulted SSI prediction model demonstrated PPV of 98%, NPV of 96%, and AUC of 0.82 (95% CI 0.81–0.83). The accuracy of the model after a 10-fold cross-validation was 94.1%. Figure 2 presents the top 30 important physician diagnostic (ICD-9) codes for prediction of SSIs that have been identified using VIM.

Stage 2: The identified top 30 physician diagnostic codes were input into the high-performance logistic regression model to identify the best parsimonious model for prediction of SSIs, using a stepwise selection approach. Table 1, Model 2 presents the final models of nine physician diagnostic codes to identify SSIs (AUC 0.85, 95% CI 0.84–0.86).

Stage 3: Risk scores for the final model of physician diagnostic codes are presented in Table 1, Model 2 [27]. Among the entire cohort, 77.8% of patients had a score of 0, 7.7% had a score of 1, and 14.5% had a score equal or greater than 2.

### Predictive modeling for physician procedure claims

We identified 2543 physician procedure claims recorded within 30 days following the surgery date. These codes then were clustered into 610 three-digit codes that were used for the further analyses.

**Fig. 3:**
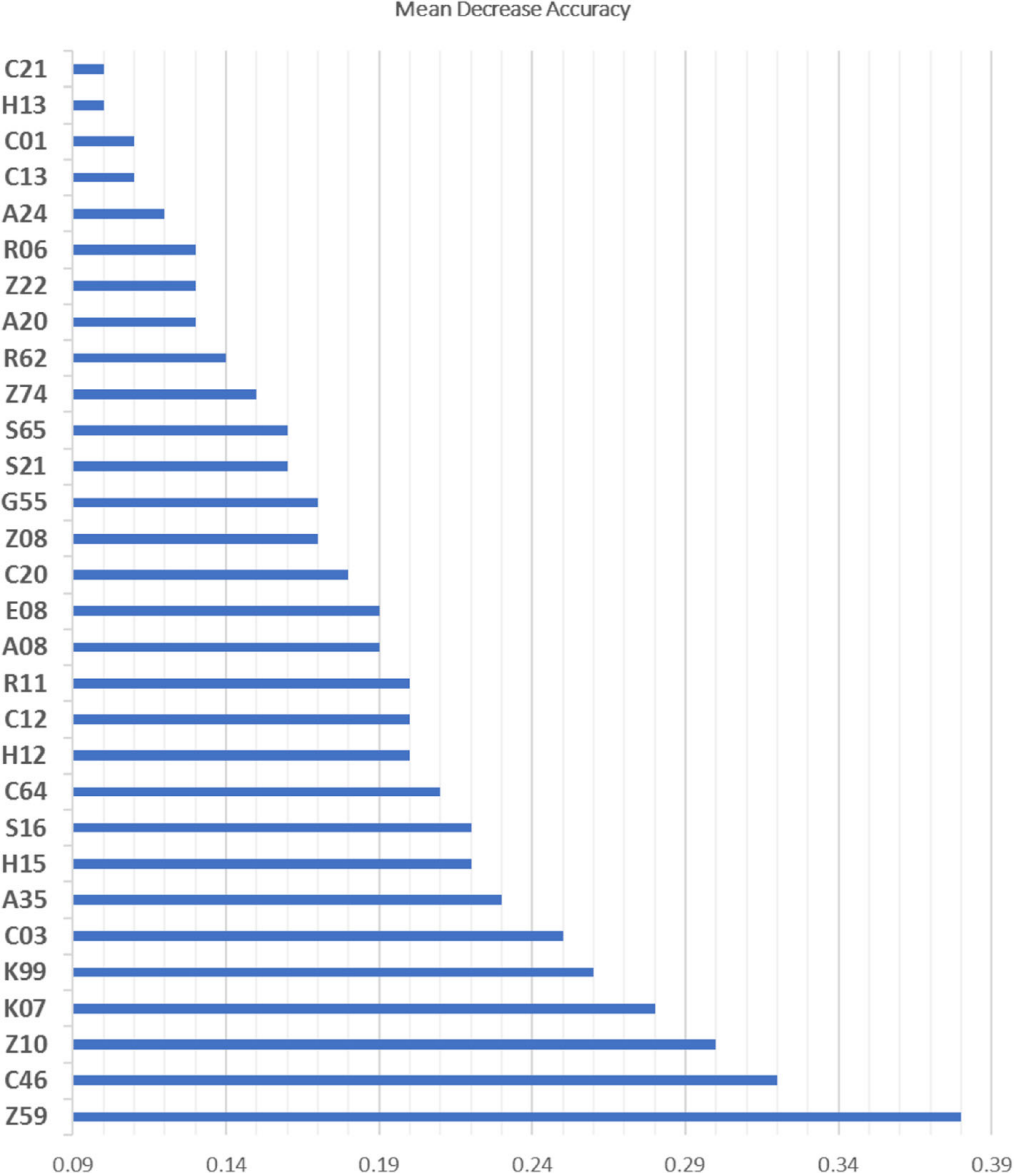
Description of the top 30 physician procedure claims to identify SSIs. Z59 - Digestive system surgical procedure; C46 - Infectious disease - non-emergency hospital in-patient services: assessment/ consultation; Z10 - Integumentary system surgical procedures: incision of abscess/ haematoma; K07 - Family practice/geriatrics acute and chronic home care supervision; K99 - Emergency department – special visit premium; C03 - General surgery, non- emergency hospital in-patient services-assessment, visits, consultations; A35 - Urology -consultations/ assessment; S16 - Digestive system surgical procedures; H15 - Family practice & practice in general - weekend and holidays: assessment/care; C64 - General thoracic surgery - non-emergency hospital in-patient services: consultation assessment; H12 - Family practice & practice in general - nights assessment and car; C12- Non-emergency hospital in-patient services: Subsequent visits by the MRP; R11- Integumentary system surgical procedures: operations of the breast; E08 - Hospital and institutional consultations/assessments by MRP; C20 - Obstetrics and gynecology - non-emergency hospital in-patient services; Z08 - Debridement of wound(s) and/or ulcer(s) extending into subcutaneous tissue, tendon, ligament, bursa and/or bone; G55- Diagnostic and therapeutic procedures, critical care; S21- Digestive system surgical procedures: rectum; S65 - Male genital surgical procedures; Z74 – Respiratory surgical procedures; R62- Musculoskeletal system surgical procedures – amputation; A20 - Obstetrics and gynecology - assessment or consultation; Z22 - Musculoskeletal system surgical procedures; R06 - Myocutaneous, myogenous or fascia-cutaneous flaps, neurovascular island transfer, transplantation of free island skin and subcutaneous flap; A24 - Otolaryngology – assessment/ consultation; C13 - Internal and occupational medicine: non- emergency hospital in-patient services; C01 - Non-emergency hospital in-patient services, subsequent visits by the MRP; H13 - Family practice & practice in general –weekdays, evenings: assessment/care; C21 – Consultations/visits anaesthesia -non-emergency hospital in-patient services

Stage 1: Given a large number of physician procedure codes (possible predictors), Random forests approach was used to identify a subset of 30 physician procedure claims that best predicts SSIs. The best misclassification rate was achieved by using 1000 classification trees and 37 variables available for splitting at each tree node. The accuracy of the Random Forests model was 94.8%. The resulted SSI prediction model demonstrated PPV of 99%, NPV of 97%, and AUC of 0.82 (95% CI 0.81–0.83). The accuracy of the model after a 10-fold cross-validation was 94.4%. Figure 3 presents the top 30 physician procedure claims that have been identified using the permutation VIM.

Stage 2: The identified top 30 physician procedure claims were input into the high-performance logistic regression model to identify the best parsimonious model for prediction of SSIs. We used a stepwise variable selection approach. Table 1, Model 3 presents the final models of 14 physician procedure claims to identify SSIs (AUC 0.84, 95% CI 0.83–0.85).

Stage 3: Risk scores for the final model of physician procedure claims are presented in Table 1, Model 3 [27]. Among the entire cohort, 55.4% of patients had a score of 0, 11.9% had a score of 1, and 44.6% had a score equal or greater than 2.

### Full model with total risk score of diagnostic and procedure codes

In the derivation cohort, the total scores of hospitalization diagnostic (ICD-10) codes, physician diagnostic (ICD-9) codes and physician procedure claims were included in the logistic regression model and adjusted for potential confounding factors, including surgical specialties, age, sex, duration of surgery, emergency case, ASA class and concurrent surgical procedures (Table 2).

**Table 2:** Full model of total risk scores for hospitalization diagnostic (ICD-10) codes, physician diagnostic (ICD-9) codes and physician procedure claims, adjusted for the study covariates

Effect	Adjusted Odds Ratio	95% Confidence interval
**Hospitalization diagnostic score**	2.12	1.91–2.20
**Physician diagnostic score**	1.88	1.75–2.02
**Physician procedure score**	1.45	1.31–1.56
**Age** < 65 years	1.74	1.40–2.16
**Log-operation duration, min**	1.52	1.30–1 .72
**Surgical specialty**		
General surgery	1.60	1.20–2.15
Gynecology	1.19	0.80–1.76
Orthopedics	0.77	0.53–1.11
Plastics	2.37	1.59–3.51
Vascular	1.75	1.12–2.68
Other	Reference	Reference
**Female**	1.18	0.96–1 .47
**Concurrent surgical procedures**		
1	1.05	0.67–1.63
2+	1.09	0.67–1.75
0	Reference	Reference
**ASA class**		
I	0.87	0.75–1.33
II	1.21	0.80–1.80
III	1.10	0.66–1.76
IV	0.32	0.04–1.03
V	Reference	Reference
**Emergent case**	0.99	0.79–1.20

**Fig. 4:**
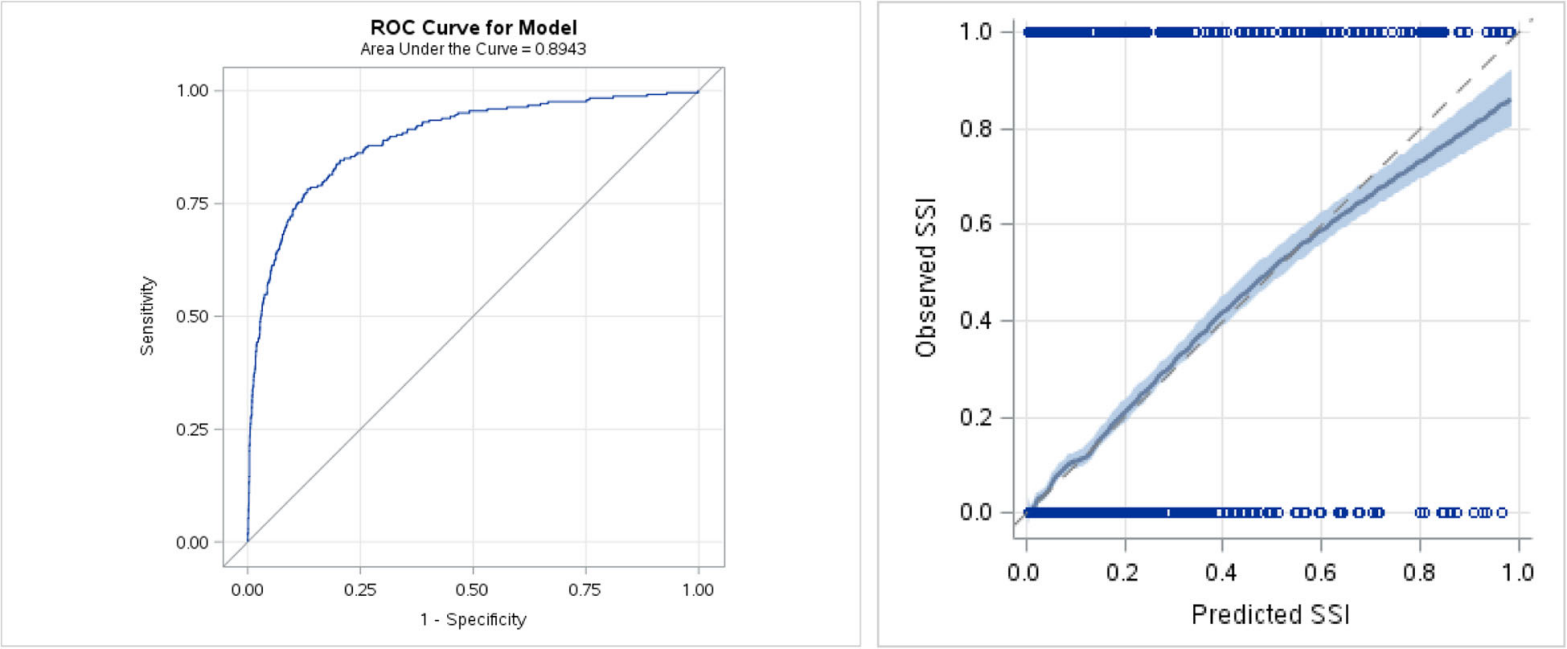
Receiver Operator Characteristics Curve (ROC curve) and ^*^calibration plot for the full model with risk scores for hospitalization diagnostic (ICD-10) codes, physician diagnostic (ICD-9) codes, and physician procedure claims, adjusted for the ^**^study covariates, in the validation cohort. ^*^In the calibration plot, the observed percentage of patients having an SSI within 30 days of surgery is plotted against the predicted SSI risk from the SSI risk model (horizontal axis). ^**^Study covariates: surgical specialties, age, sex, duration of surgery, emergency case, ASA class, concurrent surgical procedures

The full model had excellent discrimination (AUC 0.91; 95% CI, 0.90–0.92) and calibration (H-L statistics, 4.53, *p =* 0.402). The predicted probability threshold with the optimal operating characteristics [32] (e.g., the square of distance between the point (0, 1) on the upper left hand corner of ROC space and any point on ROC curve) was a predicted risk of 4% (sensitivity, 83.4%; specificity, 89.2%; PPV, 34.2%; and NPV, 99.1%). In the internal validation cohort, the full model remained strongly discriminative (AUC 0.89, 95% CI 0.88–0.90) and well calibrated (H-L statistics, 6.47, *p* = 0.487) (Fig. 4).

## Discussion

We used a 3-stage predictive modeling approach to derive and internally validate models to predict SSIs within 30 days after surgical procedure. To the best of our knowledge, this is the first study that used Machine Learning approaches to develop efficient algorithms for identifying SSIs within 30 days after surgery by use of health administrative data. The key finding of our study is that the risk of SSIs can be reliably estimated using routinely collected administrative data, including physician procedure claims, hospital (ICD-10) and physician (ICD-9) diagnostic codes. Our study results demonstrate high performance of the Random Forests algorithm for prediction of SSIs without pre-selection of possible predictors given a small number of cases. We derived a relatively small set of variables to identify postoperative SSIs, including 6 hospital diagnostic codes, 9 physician diagnostic codes, and 14 physician procedure claims.

Several studies have examined the use of administrative data to identify postoperative SSIs [6–10]. Our study findings are consistent with these studies [6, 10]. van Walraven et al. [6], for example, found that administrative data, including hospital diagnostic, emergency department visit codes and physician procedure claims, can be effectively used to identify postoperative patients with a low risk of having SSIs within 30 days of their surgical procedure. In particular, the predictive probability threshold with the optimal characteristics was a predicted risk of 5% (sensitivity, 82.1%, specificity, 85.6%, PPV, 27.7%). Additionally, Sands et al. found that [9] automated medical and claim records together can be used to screen for post discharge SSIs, but the method they used identified only 10% of procedures as possible infections.

The approach used in our study added a new contribution to the existing literature by incorporating much larger set of features as compared with the previous studies. It was possible to include all available diagnostic or procedure codes to identify SSIs in this study, because Random Forests approach is generally unaffected by the addition of irrelevant features and is robust to collinearity due to the use of subsets of random variables for tree splits. All the features included in this study were obtained from routinely collected data, and given the complex etiology of SSIs, there might be variables that would be overlooked if we used a narrower search strategy guided by a priori clinical expectations. It would be inappropriate to interpret the identified diagnostic or procedure codes as either causes or consequences of SSIs. Random Forests allows us to select variables that are influencing prediction given a small sample sizes and the extremely small ratio of samples to variable (large “*p*” and small “*n*”). If the identified important variables are consistent with clinical knowledge, there will be more confidence in the derived model as a decision support tool.

Several aspects of our study should be carefully considered. First, our study contained no information about outpatient antibiotic treatments because the Ontario health administrative data used for the study captures medication use for people over the age of 65 and who are on social assistance. Also, we did not include information about laboratory tests, because the Ontario health administrative data captures information only on outpatient laboratory tests, while laboratory tests performed during hospitalization are most important in predicting SSIs. Thus, information about antibiotic use and laboratory test could substantially improve SSI identification. Second, our study and model captured SSIs that occurred within 30 days after surgical procedure, so any SSI that occurred outside of this timeframe would have been missed. Third, our study was conducted in a single teaching hospital, providing about 90% of the major surgical operations in a catchment area of 1.2 million people. Therefore, external validation is necessary to measure model’s utility in other hospitals and geographic regions. Finally, the coding systems used in the province of Ontario might not be available in other jurisdictions. Therefore, some modifications might be required before using our models in other health regions.

## Conclusion

This study shows that health administrative data could be effectively used in identifying SSIs. Machine learning approaches have shown a high degree of accuracy in predicting postoperative SSIs and can integrate and utilize a large amount of administrative data. The results of our study are useful in advancing current and future efforts to use administrative data for patient safety surveillance and improvement. Further research should examine the use of machine learning approaches to identify SSIs, stratified by the specific types of surgical procedures.

## Supplementary Information

**Additional file 1.** Includes American College of Surgery – National Surgical Quality I mprovement Program definition of different types of SSIs: superficial, deep and orga-space.

**Additional file 2.** Provides information about the Random Forests algorithm: constructing a large collection of decision trees with controlled variation, as well as how the multiple models are normally combined by ‘voting’.

**Additional file 3.** Provides information about the data partitioning into derivation (70%) and validation (30%) samples.

**Additional file 4.** Provides descriptive statistics for patients in the study sample.

**Additional file 5.** Provides information on the derivation and validation datasets.

**Additional file 6.** Provides information on the title of the manuscript, author list and affiliations.

## Data Availability

The data that support the findings of this study are available at the Institute for Clinical Evaluative Sciences (ICES) (www.ices.on.ca/DAS), but restrictions apply for the availability of these data, which were used under license for the current study, and so are not publicly available. Data are however available from the authors upon reasonable request and with permission of ICES.

## Abbreviations

SSI: Surgical site infections
NSQIP: National Surgical Quality Improvement Program
OHIP: Ontario Health Insurance Plan
ICD: Classification of Diseases
ASA: Patient’s physical status
H-L: Hosmer-Lemeshow statistics
PPV: Positive predictive value
NPV: Negative predictive value
VIM: Variable importance measure
AOR: Adjusted odds ratio
CI: Confidence interval
AUC: Area under curve
