# Supplementary material for: Predicting postoperative surgical site infection with administrative data: a random forests algorithm
Source: BMC Med Res Methodol. 2021 Aug 28;21:179. doi: 10.1186/s12874-021-01369-9 (PMC8403439; doi:10.1186/s12874-021-01369-9)
Supplement: Supplementary file 1 — Additional file 1. Includes American College of Surgery – National Surgical Quality I mprovement Program definition of different types of SSIs: superficial, deep and orga-space. [file 12874_2021_1369_MOESM1_ESM.docx]

**Additioanl file 1. Definition of different types of SSIs**

**American College of Surgery – National Surgical Quality Improvement Program definition of different types of SSIs**

1. A superficial SSI was counted if only skin and subcutaneous tissue of the incision were involved and any of the following were noted: purulent drainage from the superficial incision; organisms isolated from an aseptically obtained culture of fluid or tissue from the superficial incision; pain, tenderness, localized swelling, redness, or heat with superficial incision being deliberately opened by the surgeon; or diagnosis of superficial incisional SSI by the surgeon or attending physician.

2. A deep incisional SSI was counted if deep soft tissues were involved and any of the following were noted: purulent drainage from the deep incision but not from the organ or organ space; a deep incision spontaneously dehisced or was deliberately opened by a surgeon when the patient had a fever, localized pain, or tenderness; an abscess or other evidence of infection involving the deep incision was found on direct examination, during reoperation, or by histopathologic or radiologic examination; or a diagnosis of deep incision SSI was made by a surgeon or attending physician.

3. An organ-space SSI was counted if: purulent drainage noted from a drain that was placed through a stab wound into the organ/space; organisms were isolated from an aseptically obtained culture of fluid or tissue in the organ/space; an abscess or other evidence of infection involving the organ/space that was found on direct examination during reoperation, or by histopathologic or radiologic examination; or diagnosis of an organ/space SSI was made by a surgeon or attending physician.
