# Supplementary material for: Predicting postoperative surgical site infection with administrative data: a random forests algorithm
Source: BMC Med Res Methodol. 2021 Aug 28;21:179. doi: 10.1186/s12874-021-01369-9 (PMC8403439; doi:10.1186/s12874-021-01369-9)
Supplement: Supplementary file 2 — Additional file 2. Provides information about the Random Forests algorithm: constructing a large collection of decision trees with controlled variation, as well as how the multiple models are normally combined by ‘voting’. [file 12874_2021_1369_MOESM2_ESM.docx]

**Additional file 2. Random Forests algorithm**

**
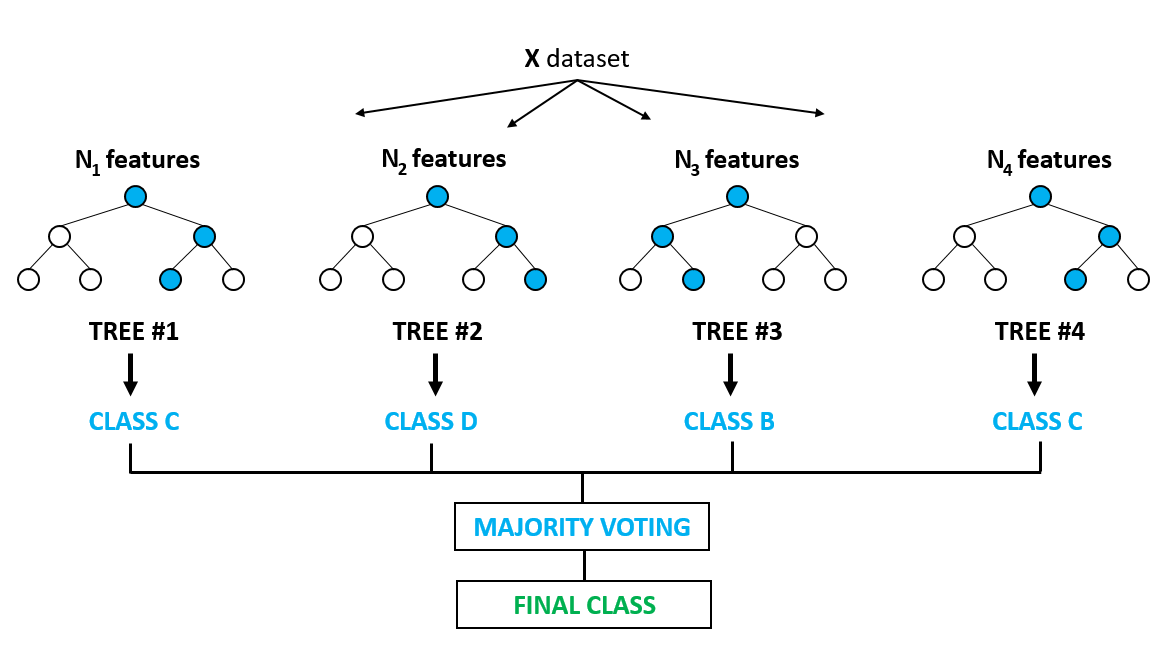
**

Bootstrapping is an uncertainty modelling step that generates modified versions of the training set over which trees will be grown and ensemble predictions averaged. Trees are grown using binary portioning. Once a node is split on the best eligible splitter, the process is repeated in its entirety on each child node. A new list of eligible predictors is selected at random for each node. When multiple models are generated, they are normally combined by ‘voting’. For every record, the proportion of votes for each class (variable) represents the probability of class membership. Winner is the class with the most votes.
