# Supplementary material for: Predicting postoperative surgical site infection with administrative data: a random forests algorithm
Source: BMC Med Res Methodol. 2021 Aug 28;21:179. doi: 10.1186/s12874-021-01369-9 (PMC8403439; doi:10.1186/s12874-021-01369-9)
Supplement: Supplementary file 3 — Additional file 3. Provides information about the data partitioning into derivation (70%) and validation (30%) samples. [file 12874_2021_1369_MOESM3_ESM.docx]

**Additional file 3. Data partitioning**

**Original dataset**

n=14.351

**Validation Partition**

n=4,305

**Derivation Partition**

n=10,046

**Out of Bag**

(1/3 of derivation set)

**Train data**

(2/3 of derivation) **set)**
