# Supplementary material for: Predicting postoperative surgical site infection with administrative data: a random forests algorithm
Source: BMC Med Res Methodol. 2021 Aug 28;21:179. doi: 10.1186/s12874-021-01369-9 (PMC8403439; doi:10.1186/s12874-021-01369-9)
Supplement: Supplementary file 4 — Additional file 4. Provides descriptive statistics for patients in the study sample. [file 12874_2021_1369_MOESM4_ESM.docx]

**Additional file 4. Description of the study cohort**

| **Characteristic** | **Cohort with SSI**  **n=795** | **Cohort without SSI**  **n=13,556** |
| --- | --- | --- |
| **Age** | | |
| Mean (SD) | 58.1 (16.6) | 56.6 (17.5) |
| ≥65 | 295 (37.1) | 4,769 (35.2) |
| **Sex,** n (%) | | |
| Female | 461 (58.0) | 7,624 (56.2) |
| **Operation time (min)** |  |  |
| Mean (SD) | 200.0 (144.6) | 114.4 (94.4) |
| **Surgical specialty,** n (%) | | |
| General surgery | 400 (50.3) | 3,052 (22.5) |
| Gynecology | 69 (8.7) | 1,297 (9.6) |
| Orthopedics | 69 (8.7) | 4,203 (31.0) |
| Plastics | 69 (8.7) | 911 (6.7) |
| Vascular | 64 (8.1) | 806 (6.0) |
| Other | 124 (15.6) | 3,287 (24.2) |
| **Emergent case**, n (%) | | |
| Yes | 173 (21.8) | 2,337 (17.2) |
| **Concurrent procedures**, n (%) | | |
| 0 | 692 (87.0) | 13,233 (97.6) |
| 1 | 51 (6.4) | 189 (1.4) |
| 2+ | 52 (6.6) | 134 (1.0) |
| **ASA score, n (%)** | | |
| I | 35 (4.4) | 1,621 (12.0) |
| II | 160 (20.1) | 4,821 (35.6) |
| III | 471 (59.2) | 5,812 (42.9) |
| IV | 125 (15.7) | 1,248 (9.2) |
| V | <=6 (0.5) | 54 (0.4) |
