# Supplementary material for: Predicting postoperative surgical site infection with administrative data: a random forests algorithm
Source: BMC Med Res Methodol. 2021 Aug 28;21:179. doi: 10.1186/s12874-021-01369-9 (PMC8403439; doi:10.1186/s12874-021-01369-9)
Supplement: Supplementary file 5 — Additional file 5. Provides information on the derivation and validation datasets. [file 12874_2021_1369_MOESM5_ESM.docx]

**Additional file 5. Description of derivation and validation sets**

| **Characteristic** | **Derivation set**  **n=10,046** | **Validation set**  **n=4,305** |
| --- | --- | --- |
| **SSI,** n (%) | | |
| Yes | 556 (5.5) | 239 (5.5) |
| **Age** | | |
| Mean (SD) | 56.6 (47.5) | 56.9 (17.1) |
| **Sex,** n (%) | | |
| Female | 5,660 (56.5) | 2,401 (55.9) |
| **Operation time (min)** | | |
| Mean (SD) | 119.2 (99.6) | 119.4 (100.3) |
| **Surgical specialty,** n (%) | | |
| General surgery | 2,408 (24.6) | 1,037 (24.1) |
| Gynecology | 958 (9.6) | 404 (9.4) |
| Orthopedics | 3,003 (30.3) | 1.265 (29.5) |
| Plastics | 672 (6.7) | 301 (7.0) |
| Vascular | 595 (5.9) | 274 (6.4) |
| Other | 2,385 (23.8) | 1,014 (23.6) |
| **Emergent case**, n (%) | | |
| Yes | 1,751 (17.5) | 759 (17.7) |
| **Concurrent procedures**, n (%) | | |
| 0 | 9,740 (97.2) | 4,150 (96.6) |
| 1 | 152 (1.5) | 88 (2.0) |
| 2+ | 129 (1.3) | 57 (1.3) |
| **ASA score, n (%)** | | |
| I | 1,167 (11.6) | 472 (11.1) |
| II | 3,476 (34.7) | 1,491 (34.7) |
| III | 4,372 (43.6) | 1,904 (41.3) |
| IV | 963 (9.6) | 408 (9.5) |
| V | 43 (0.4) | 15 (0.4) |
