# Supplementary material for: Predicting postoperative surgical site infection with administrative data: a random forests algorithm
Source: BMC Med Res Methodol. 2021 Aug 28;21:179. doi: 10.1186/s12874-021-01369-9 (PMC8403439; doi:10.1186/s12874-021-01369-9)
Supplement: Supplementary file 6 — Additional file 6. Provides information on the title of the manuscript, author list and affiliations. [file 12874_2021_1369_MOESM6_ESM.docx]

**Additional file 6. Title page**

**Predicting postoperative surgical site infection with administrative data: a Machine Learning algorithm**

Yelena Petrosyan MD MPH PhD^1^

Kednapa Thavorn MPharm PhD^1,2,3*^

Glenys Smith MSc^3^

Malcolm Maclure ScD^4^

Roanne Preston MD FRCPC^4^

Carl van Walrevan MD, MSc^1,2,3^

Alan J. Forster MD FRCPC MSc^1,3,5^

1. Clinical Epidemiology, Ottawa Hospital Research Institute, 1053 Carling Ave, Ottawa, Ontario, K1Y 4E9, Canada
2. School of Epidemiology and Public Health, University of Ottawa, 75 Laurier Ave E, Ottawa, Ontario, K1N 6N5, Canada
3. Institute for Clinical and Evaluative Sciences, 1053 Carling Ave, Ottawa, Ontario, K1Y 4E9, Canada
4. Department of Anesthesiology, Pharmacology and Therapeutics, University of British Columbia, Vancouver, British Columbia, V6T 1Z4, Canada
5. Department of Medicine, University of Ottawa, 75 Laurier Ave E, Ottawa, Ontario, K1N 6N5, Canada

***Corresponding author:**

Kednapa Thavorn, MPharm, PhD

The Ottawa Hospital - General Campus

501 Smyth Road, PO Box 201B,

Ottawa, ON, K1H 8L6

Phone: 613-737-8899 ext 72330

Email: [kthavorn@ohri.ca](mailto:kthavorn@ohri.ca)
